# Marine Mycobiomes Colonize Mediterranean Sponge Hosts in a Random Fashion

**DOI:** 10.1007/s00248-025-02523-2

**Published:** 2025-04-10

**Authors:** Valerio Mazzella, Geoffrey Zahn, Antonio Dell’Anno, Laura Núñez Pons

**Affiliations:** 1 Department of Integrative Marine Ecology (EMI), Stazione Zoologica Anton Dohrn, Ischia Marine Centre, Ischia, Naples, 80077 Italy; 2 NBFC, National Biodiversity Future Center, Piazza Marina 61, Palermo, 90133 Italy; 3 Biology Department, Utah Valley University, 800 W University Parkway SB243c, Orem, UT 84058 USA; 4 Department of Life and Environmental Sciences, Polytechnic University of Marche, Via Brecce Bianche, Ancona, 60131 Italy; 5 Department of Integrative Marine Ecology (EMI), Stazione Zoologica Anton Dohrn, Villa Comunale, Naples, 80121 Italy

**Keywords:** Mycobiomes, Mediterranean sponges, Marine fungi, Porifera, Microbial symbiosis

## Abstract

Marine sponges are widespread, sessile, filter-feeding animals, known for living in association with complex prokaryotic communities structured by host species. Though marine fungi are ubiquitous across marine environments, little is known about sponge-associated fungal communities (mycobiome). Indeed, aside from a few studies based on the isolation of fungal strains for biotechnological purposes, little information is available to understand the diversity and structure of sponge mycobiome. Here, a metabarcoding approach based on the ITS1 marker was applied to examine the structure and composition of fungal communities associated with four Mediterranean sponges. The species: *Petrosia ficiformis*, *Chondrosia reniformis*, *Crambe crambe*, and *Chondrilla nucula* were analyzed along with the surrounding seawater, revealing *Aspergillus* (1–56%), *Cladosporium* (1–75%), *Malassezia* (1–38.5%), and *Pennicillium* (1.5–36%) as the most represented fungal genera. Our data showed high intra-specific variability and no clear core mycobiome within each of the sponge species host, suggesting stochastic and perhaps transient community membership. This study sheds light on one of the most abundant yet least understood components of the marine ecosystem. Unraveling the dynamics of fungal interactions within sponge holobionts is essential to advance our understanding of their ecological roles and functions. By addressing the enigmatic nature of sponge-associated fungi, this research opens new avenues for exploring their contributions to marine ecosystems and resolving the many unanswered questions in this field.

**Supplementary Information:**

The online version contains supplementary material available at 10.1007/s00248-025-02523-2.

## Introduction

Sponges (phylum Porifera) are complex and diverse filter feeders that play crucial roles in marine ecosystems by providing habitat, food, and shelter to a wide range of organisms [1]. As model holobionts, they harbor dense, functional microbial communities that can make up a significant proportion of the host’s weight [2, 3]. Associated microbes furnish their hosts with essential nutrients and energy resources, assist in waste management, and produce secondary metabolites that can help deter predators, prevent fouling, or compete for space [4–6]. Additionally, these microbiomes play important roles in carbon, nitrogen, and sulfur biogeochemical cycling [2, 7]. The taxonomic composition of the associated prokaryotic microbiota is generally species-specific and has a strong ecological relevance [4, 7]. The quantitative importance of the prokaryotic communities (bacteria and archaea) associated with sponges has been deeply studied over the past decade prompting researchers to divide Porifera hosts in two major groups: high microbial abundance (HMA) and low microbial abundance (LMA) species [8–10].

Fungi are widespread organisms in all marine environments, from surface waters to deep-sea sediments [11, 12], and their biodiversity seems to be structured more by local environmental variables than large-scale biogeographic factors [13]. Marine fungi have been found living on or inside other host such as algae, corals, sponges, and even other fungi [14]. Despite their conspicuous presence and distribution, marine fungi, especially those living in association with invertebrates, still remain understudied [15, 16]. In particular, quantitative data (i.e., fungal cell densities) are absent. Since free-living marine fungal cells appear in roughly three orders of magnitude lower concentrations than bacteria (10^3^ versus 10^6^) [12, 17], without a selection mechanism during filter-feeding, fungal cells in sponges are expected to be in much lower densities than bacteria.

Fungi associated with invertebrates can have varying relationships to their holobiont hosts, spanning a continuum of commensal, parasitic, or mutualist roles [16]. Their ecological role can be complex and can depend on different factors, such as fungal origin (terrestrial or strictly marine), host health, environmental variability, and the co-occurrence of other microorganisms within the host [16]. Most of the documented examples of marine fungal symbioses are in lichens, where they are closely associated with macroalgae or cyanobacteria. In these interactions, fungi play a pivotal role providing a suitable habitat for their partners (providing water, fertilizing with micronutrients, offering micro-niches), while exploiting photosynthetically fixed carbon and other products for nourishment [18–20]. These types of fungal associations have been shown to work also in artificial microbial consortia, enhancing the production of secondary metabolites [21]. Fungal strains have been found associated with some species of marine macroalgae (mycophycobioses), assisting in seaweed resistance to environmental changes [22]. One of the few examples of invertebrate-fungal associations described are certain endolithic coral fungi, which have been proposed to be involved mainly in the nitrogen cycle within the coral holobiont [23, 24]. Fungal interactions, however, are not always positive, and there are cases of fungal diseases reported in corals and sponges [25–27]. 

The majority of studies involving sponge-fungal associations use culture-based approaches, which allow the isolation and identification of some fraction of fungal strains. These investigations are chiefly bioprospecting, seeking for the discovery of new fungal derived metabolites with biotechnological potential [28–30]. The unculturable fungal biosphere has been more recently investigated since the emergence of omics techniques, allowing the scientific community to examine the diversity and composition of marine mycobiomes [31]. However, a major constraint in using metabarcoding to study fungal-invertebrate relationships is the prevalence of host DNA co-amplification along with the fungal DNA [15, 32, 33]. This is particularly limiting when using the ITS1 gene as a barcode marker [15], which is the most informative for fungal diversity [34]. The extent of undesirable host DNA co-amplification can vary greatly across species, ranging from undetectable to over 99% of the total reads, as reported in most sponges and corals [15]. This restraint is for the most part generated by the difficulty of developing fungal-specific primers able to attach in discriminative priming sites at the flanking ends of the ITS1 [35]. Particularly in corals and sponges, these sites seem to be highly conserved and phylogenetically closely related to fungi [36]. Several attempts have tried to circumvent these co-amplification issues, with the design of new primers, restriction enzymes, and genetic probes, with limited success [32, 35]. Furthermore, selecting a curated and well-annotated reference database is crucial to have a fitting taxonomic annotation [37], considering that some of the existing databases are focused on terrestrial fungi and do not have many marine fungal representatives [38]. All these constraints often impede the analysis of fungal communities in Porifera holobionts, making this field challenging and full of gaps in knowledge [14, 15, 35].

In our research, we considered four sponge holobionts widespread in the Mediterranean Sea with different ecological traits: *Petrosia ficiformis* (Poiret, 1798), *Chondrosia reniformis* Nardo 1847, *Chondrilla nucula* Schmidt, 1862 are HMA species, and *Crambe crambe* (Schmidt, 1862) is LMA*.* These species are considered to be mostly heterotrophic [39], except for *C. nucula*, which from experimental and observational hints has been proposed to rely on photosymbionts (Cyanobacteria) for nourishment [39–41]. Prokaryotic communities associated with these sponge hosts have been widely investigated, revealing a consistent species-specific trend [8–10, 39, 42, 43]. Previous investigations have only provided limited and incidental information on the presence of fungi in Mediterranean Porifera. However, some information confirms the presence of sponge-associated fungi. For example, vertically transmitted symbiotic yeasts were reported in sponges in genus *Chondrilla*, through microscopic observations [44], and the culturable mycobiome was assessed in the sponge *C. crambe* [45], along with several other surveys focusing on sponge associated fungi for biotechnological applications [29, 45–48]. Here we present an analysis and comparison of the mycobiome community compositions of Mediterranean sponges in order to provide baseline knowledge of sponge-associated fungi and to facilitate future ecological research on sponge-fungal interactions.

## Materials and Methods

### Sponge Collection

Samples were collected around the island of Ischia, Naples (Southern Thyrrhenian, Mediterranean Sea) on the spring 2018. Sponge pieces (~ 10 cm^3^) of the target species *Petrosia ficiformis* (*n* = 10), *Crambe crambe* (*n* = 15), *Chondrilla nucula* (*n* = 10), and *Chondrosia reniformis* (*n* = 15) were collected along with ambient seawater samples in 10-L containers (*n* = 9) in spring 2018 by scuba diving at 2–5 m depth around the island from three sites with similar characteristics: Castello Aragonese (CCO; 40°43′55.9″N–13°57′52.9″E), Grotta Mago external (GF; 40°42′41.6″N–13°57′51.4″E) and Sant’Anna (SA; 40°43′36.5″N–13°57′43.4″E). Sponge samples were stored at 4 °C, in zip bags filled with their surrounding seawater, and brought immediately to the laboratory for processing. Seawater samples were filtered with Gelman 47-mm magnetic filter funnel (Gelman Sciences, MI, USA) system through 0.22-μm pore size filters (Millipore MFTM-Membrane), and filters were stored at − 80 °C. Pieces of 0.5 cm^3^ from all sponge samples were snap-frozen in liquid nitrogen and stored at − 80 °C until processed.

### DNA Extraction, Amplification, and Sequencing

DNA from frozen sponge samples and seawater filters was extracted using QIAGEN PowerSoil Pro Kit, following manufacturer’s instructions. The quantity and quality of the extracted DNA were assessed through a Thermo Scientific Nanodrop™ 1000. The ITS1 region of the internal-transcribed spacer (ITS) was amplified using the universal fungal primers ITS1F (5′-CTTGGTCATTTAGAGGAAGTAA- 3′) and ITS2R (5′-GCTGCGTTCTTCATCGATGC- 3′) [49, 50] following the Illumina protocol with amplification reaction containing the following: 2.5 µL DNA (5 ng/µL), 5 µL forward primer (1 µM), 5 µL reverse primer (1 µM), and 12.5 µL KAPA HiFi HotStart ReadyMix, for a total volume of 25 µL. PCRs were performed in a Veriti™ 96-Well Thermal Cycler under the following conditions: 95 °C for 3 min followed by 25 cycles of 95 °C for 30 s, 55 °C for 30 s, and 72 °C for 30 s and a final elongation performed at 72 °C for 5 min followed by a 4 °C hold. To avoid host ITS co-amplification, a PCR clamping approach [51] was used in which 1 µL peptide nucleotide acid (PNA) (10 mM) was added to the master mix (according to methods from Núñez-Pons, unpublished data). PCR products were amplified to attach Illumina sequencing adapters using the Nextera XT Index Kit and purified, according to Illumina protocol.

Purification was performed with AMPure XP beads to remove potential primers and primer-dimer fragments following manufacturer’s instruction. Briefly PCR products were added and mixed with AMPure XP (1.2 µL of AMP beads solution every µL of PCR product) left a room temperature for 5′ and then placed in a magnetic rack to be washed twice with 200 µL of EtOH 70%. Finally, the beads linking the DNA were dried at room temperature, and the DNA was eluted in 12 µL of sterile ultrapure water.

After the purification step, DNA libraries underwent fluorometric quantification and were then normalized and pooled. Sequencing was performed using a MiSeq Illumina sequencer with MiSeq Reagent Kit v3 (300 cycle), targeting a sampling depth of 50,000 reads per sample. Library preparation and sequencing were conducted by Personal Genomics Ltd (http://www.personalgenomics.it).

### Bioinformatic Analyses

Bioinformatics analyses were performed according to methods from Wainwright and co-authors [52]. Briefly, the ITS1 region was isolated from the sequence reads using the ITSxpress tool [53]. After extraction, the reads underwent quality screening, with reverse reads being discarded as this approach has been shown to yield better results [54]. The DADA2 package in R was then used to process the forward reads, filtering out any reads containing uncalled bases or with a maximum expected error value of 2 (more details on these filtering parameters can be found at https://benjjneb.github.io/dada2/). The quality-filtered reads were then utilized to estimate and correct sequencing errors and to eliminate chimeras and singletons, using the DADA2 package. The resulting amplicon sequence variants (ASVs) were categorized taxonomically using the RDP Classifier algorithm against a custom database composed of the UNITE database (v. 1.12.2017) and a customized collection of outgroups that included Porifera and Anthozoa ITS1 sequences [37] (available at: https://github.com/gzahn/Enhalus_Fungi/tree/master/Taxonomy). Any non-fungal sequences were filtered and excluded from the analysis (see Fig. S1). The remaining ASVs that were taxonomically assigned as fungi were used in all subsequent analyses, which were conducted using the phyloseq R package [55]. Raw ASV counts were normalized by read count for all downstream analyses, except for differential abundance testing, which incorporated library size as a model term. More details for these steps, including all analysis code, are available in an archived GitHub repository [56].

### Alpha and Beta Diversity Analyses

Alpha diversity analyses of fungal communities associated with the four sponge species and present in the seawater were computed using two metrics, observed ASV richness and Shannon diversity index [57]. For beta diversity analyses, Bray–Curtis and Jaccard resemblance matrices were applied [58, 59]. Alpha and beta diversity analyses were run on normalized ASV tables [60] and visualized as PCoA ordinations. All analyses were conducted in R version 4.4.2 using the “phyloseq” and “phylosmith” packages [55, 61] unless otherwise noted.

### Statistical Analyses

Differences in alpha diversity values of fungal communities (observed ASVs and Shannon index) among sponge holobionts and the seawater were tested using the analysis of variance test (ANOVA). Permutational multivariate analysis of variance (PERMANOVA, 9999 permutations) was used to test for differences in community structure across the fungal communities [62]. All significance thresholds for statistical tests were set to *P* value < 0.05: i.e., FDR (false discovery rates) adjusted *p*-value. Similarity percentage analysis (SIMPER) [63] was run on PRIMER 6 + software [64] to calculate turnover diversity and to visualize community similarity within and between sampling groups. Co-occurrence networks at the ASV and the genus taxonomic levels were computed and visualized using the Gephi software v0.9.2 and Cytoscape 3.10.2 [65].

## Results

### Alpha and Beta Diversity of Fungal Communities

**Fig. 1:**
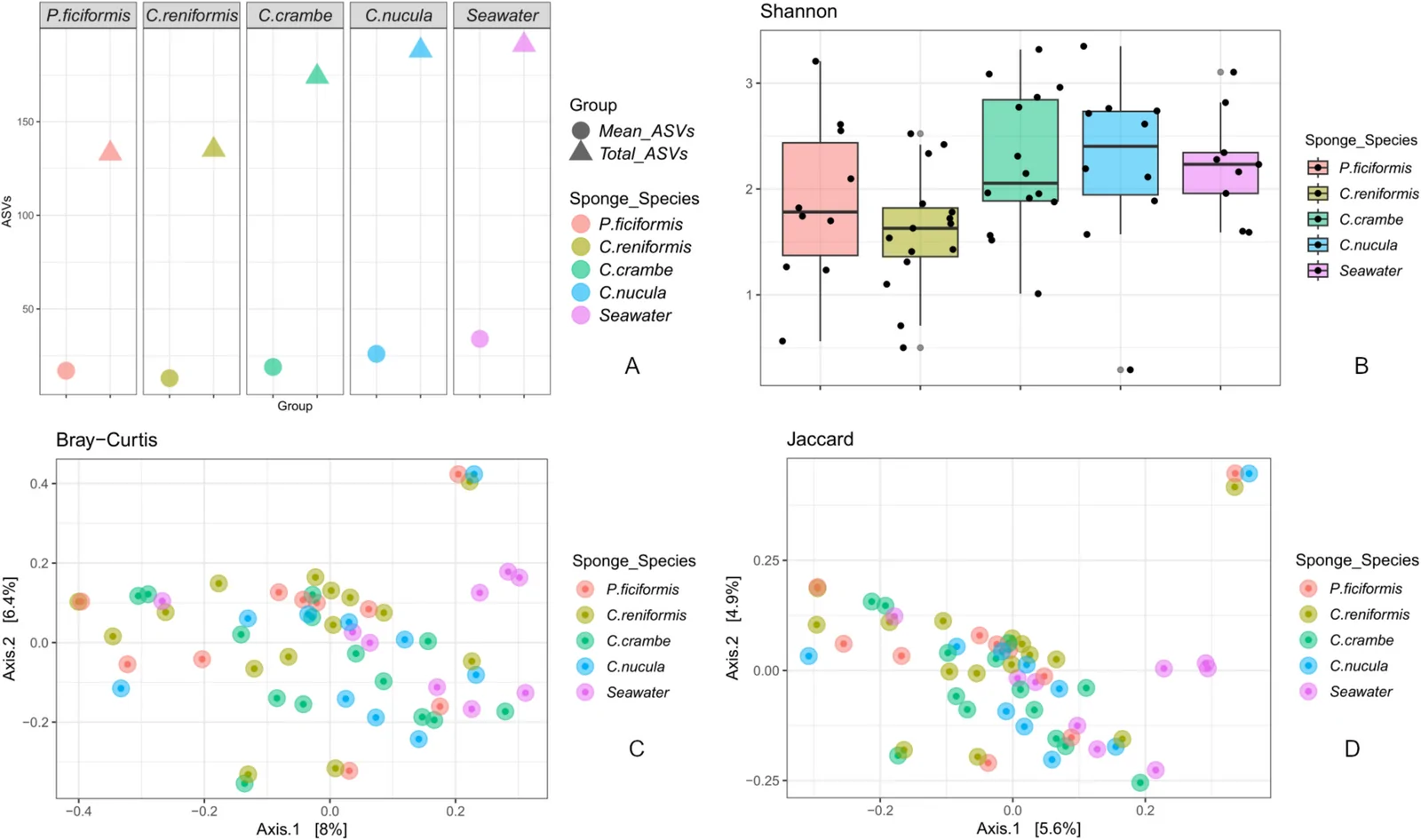
Alpha and beta diversity. **A** Mean and total observed ASVs found in each sponge species. **B** Alpha diversity boxplot based on Shannon index. **C**, **D** PCoA ordinations based on the **C** Bray Curtis resemblance and **D** Jaccard dissimilarity of the mycobiome associated with the four sponge species. Each dot represents a sample, while each sponge species is highlighted with a different color. In both of the PCoA plots, the variance explained by the two first components was lower than 15%

A total of 217,662 reads yielded 163,442 untargeted reads (70%), while fungal reads accounted for 54,220 (25%). The remaining 5% of sequences were assigned to other kingdoms and were discarded. After filtration, 42,620 fungal sequence reads remained across 58 samples, resulting in 478 unambiguous fungal ASVs. Observed ASV number ranged from 2 (*C. reniformis)* to 73 (recorded in *C. nucula*), with inconsistent numbers of ASV per sample (Fig. 1A). *Petrosia ficiformis* (mean = 17 ± 6.46; total = 133) and *C. reniformis* (mean = 13 ± 3.94; total = 135) were characterized by a lower number of total ASVs compared with *C. crambe* (mean = 19 ± 3.62; total = 174), *C. nucula* (mean = 26 ± 7.59; total = 188), and the seawater (mean = 34 ± 5.07; total = 191). This trend was also consistent considering values of Shannon alpha diversity (Fig. 1B). Differences in both alpha diversity metrics between sponge taxa were not statistically significant (ANOVA test; *p*-value > 0.05; Table S1 A-B).

Beta diversity analyses revealed scattered sample distributions (Fig. 1C, D) and poor clustering, with no statistical significance (PERMANOVA, *p*-value > 0.05; Table S2).

### Composition of the Sponges’ Associated Mycobiome

**Fig. 2:**
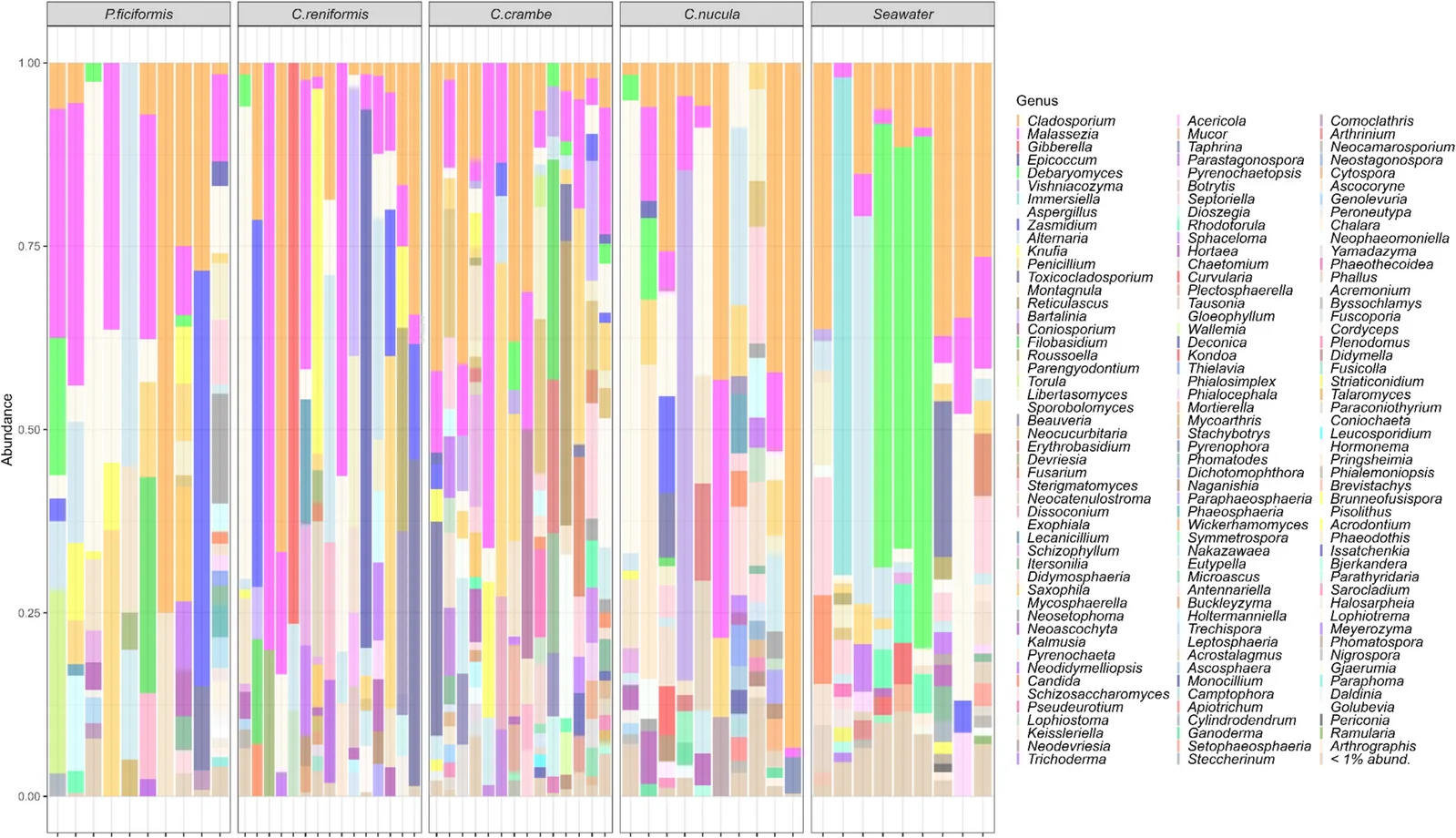
Taxonomic composition of the mycobiome associated with the four Mediterranean sponge at the genus level. Unknown assignments were removed from this visualization, and their proportion is reported in Fig. S1. Samples were divided by sponge species, and each bar represents a single sample. Fungal genera below 1% in relative abundance were clustered together as “ < 1% abund.” category

More than 50% of ASVs were not unambiguously matched to fungal taxa. The relative contribution of these non-fungal ASVs varied across host species as follows: 68% in *C. nucula*, 77% in *C. reniformis*, 56% in *C. crambe*, and 57% in *P. ficiformis* (Fig. S1). The taxonomic annotations of the filtered ASV table allowed the identification of a total of 5 phyla, 19 classes, 58 orders 129 families, and 204 genera of fungi (Fig. 2; Table S3).

*P. ficiformis* was characterized by the lowest number of different fungal ASVs, classified in 2 phyla, 11 classes, 31 orders, 53 families, and 73 genera. The most abundant fungal genus found in association with this sponge was *Cladosporium*, which ranged ~ 1–75%. Other five relevant genera were *Aspergillus* (1–56%)*, Zasmidium* (1–63%), *Alternaria* (1–55%)*, Malassezia* (1–38.5%), and *Penicillium* (1.5–36%). *C. reniformis* showed a mycobiome composed by 2 phyla, 12 classes, 26 orders, 52 families, 76 genera. *Malassezia* was the most abundant genus ranging ~ 1–80%, followed by *Gibberella* (76%), *Epicoccum* (76%), *Cladosporium* (1–66%), *Aspergillus* (1–64%), and *Zasmidium* (1–50%). *Crambe crambe*–associated mycobiome resulted in 5 phyla, 16 classes, 38 orders, 72 families, and 99 genera. *Malassezia* was also the prevalent genus 2–66% in this species, along with *Pennicillium* (1–45%), *Cladosporium* (2–41%), *Reticulascus* (38%), *Coniosporium* (1–31%), and *Filobasidium* (1–30%). *Chondrilla nucula* was characterized by the highest number of fungal ASVs compared with the other sponges, which were distributed in 2 phyla, 13 classes, 35 orders, 69 families, and 103 genera. The most abundant genus was *Cladosporium*, ranging 1–93%, followed by *Vishniacozyma* (1–69%), *Aspergillus* (1–61%), *Montagnula* (42%), *Malassezia* (1–35%), and *Alternaria* (1–24%).

The fungal composition found in the surrounding seawater was the richest in terms of ASV number, reporting 3 phyla, 16 classes, 39 orders, 77 families, and 112 genera of which *Debaryomyces* (1–69%), *Immersiella* (0–67%), *Alternaria* (1–52%), *Aspergillus* (1–39%), *Cladosporium* (6–37%), and *Toxicocladosporium* (1–21%) were the most abundant genera (see Table S4 for details on other taxonomical groups).

**Fig. 3:**
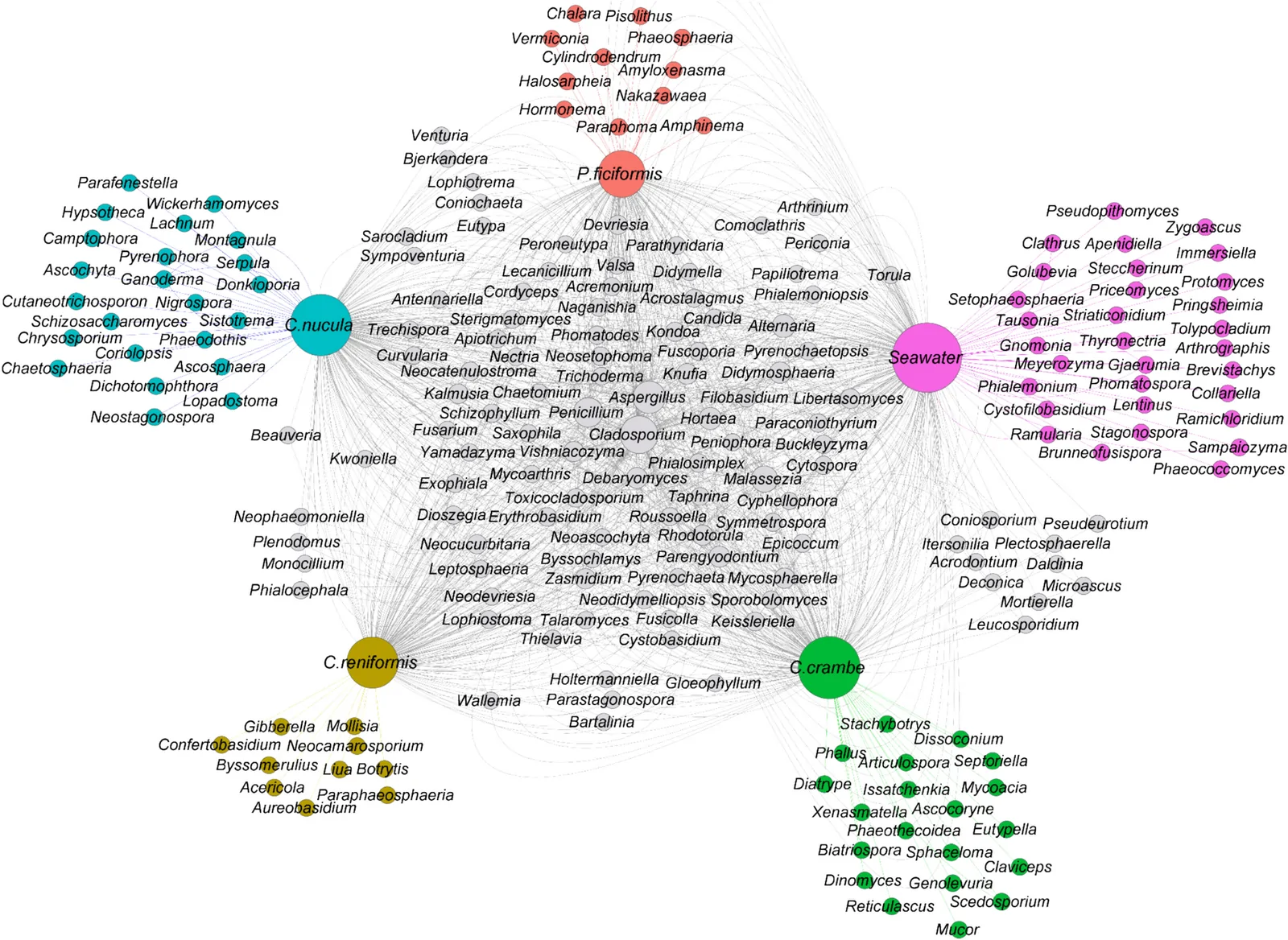
Network analysis of fungal genera sharing among the four sponge species and the seawater. Each node represents a single fungal genus. Each sponge species and the seawater were depicted in diverse colors. The size of each node is proportional to the average of its relative abundance on the overall dataset

No core ASVs were observed among the four sponge species (no ASVs shared in 100% of the samples), both considering the overall dataset, or within each sponge species. In fact, the majority of ASVs were exclusive to each sponge species (Fig. S2). *Chondrosia reniformis* showed the lowest number of exclusive ASVs (39), while *C. crambe*, *C. nucula*, and *P. ficiformis* showed respectively 78, 67, and 42 exclusive ASVs (Fig. S3). The fungal community in the seawater revealed the highest number of exclusive ASVs (88). *Crambe crambe* and *C. nucula* shared the highest number of ASVs with the seawater (70) followed by *P. ficiformis* (60) and *C. reniformis* (52). At the genus level, *C. nucula* showed the highest number of exclusive fungal genera (23) followed by *C. crambe* (20), *P. ficiformis* (11), and *C. reniformis* (10), while the fungal community belonging to the seawater compartment revealed 31 exclusive genera (Fig. 3). Among the fungal genera identified, only 31 were shared among the four sponge species and the surrounding seawater, being not present in every sample (Table S4).

Only three fungal genera (*Trichoderma*, *Yamadazyma*, *Apiotrichum*) were found in all four sponge taxa (Fig. 3, Table S4). *Petrosia ficiformis* shared two fungal genera with *C. reniformis* (*Neoascochyta*, *Kwoniella*), two with *C. crambe* (*Coniosporium*, *Torula*) and four with *C. nucula* (*Lophiotrema*, *Bjerkandera*, *Venturia*, *Coniochaeta*). *Chondrosia reniformis,* in turn, shared three fungal genera with *C. crambe* (*Bartalinia*, *Parastagonospora*, *Holtermanniella*) and one with *C. nucula* (*Beauveria*). Finally, *C. crambe* and *C. nucula* shared six fungal genera with each other (*Phialocephala*, *Plenodomus*, *Neophaeomoniella*, *Wallemia*, *Chaetomium*, *Monocillium*) (Fig. 3, Table S4). *Crambe crambe* shared the highest number of fungal genera with the seawater (*Pseudeurotium*, *Microascus*, *Plectosphaerella*, *Deconica*, *Itersonilia*, *Acrodontium*, *Daldinia*, *Mortierella*, *Leucosporidium*), followed by *C. nucula* (*Eutypa*, *Phialemoniopsis*, *Sarocladium*, *Papiliotrema*, *Sympoventuria*), *C. reniformis* (*Gloeophyllum*, *Peniophora*, *Cyphellophora*, *Fusicolla*), and *P. ficiformis* (*Periconia*, *Arthrinium*, *Comoclathris*) (Fig. 3, Table S4).

SIMPER analysis revealed a high dissimilarity (> 90%) of the fungal composition among the different sponge species, along with a high intraspecific variability. The similarity within each sponge species was indeed very low (3.3%, 3.6%, 5.9%, and 4.4% in *P. ficiformis*, *C. reniformis*, *C. crambe*, and *C. nucula*, respectively).

## Discussion

Sponges are rich repositories of microbial assemblages, especially for bacterial and archaeal taxa [2, 7], and were expected to harbor relevant repositories of fungi as well [14, 15]. The primary finding of our research was an extensive intraspecific fluctuation in diversity and taxonomic composition of associated fungi across the four sponge species analyzed. These results suggest that fungal community assembly is stochastic and/or transient in the observed sponge species.

*Aspergillus*, *Cladosporium*, *Malassezia*, *Pennicillium*, *Toxicocladosporium*, *Zasmidium*, *Alternaria*, and *Parengyodontium* were the most noteworthy genera reported in the present study, given their high relative abundance and reported habitat plasticity. All these fungal genera have been previously documented in the marine environment. *Aspergillus* and *Penicillium* have been found to be associated with sea urchins [66], while *Cladosporium* and *Penicillium* have also been linked to deep-sea coral gardens [67]. Notably, the genus *Malassezia* has been found to be associated with stony corals and found in various other marine compartments [33]. Other relevant fungal genera related to our work, such as *Candida* and *Rhodotorula*, were identified in association with marine organisms inhabiting hydrothermal vents [68]. Among the abovementioned fungal genera, *Aspergillus*, *Cladosporium*, *Malassezia*, *Pennicillium*, *Parengyodontium*, and *Alternaria* were previously found to be isolated from other sponge species from different seas [29, 46, 69–71]. These associations, however, are not always positive, being a member of the genus *Aspergillus* the cause of a fatal infection for the marine sponge *Chondrosia reniformis* [27]. In contrast, the genus *Zasmidium*, to the best of our knowledge, was never mentioned to be associated with marine sponges being isolated from other invertebrates or plants [72, 73]. All these fungal genera were also found in deep sea sediments and seem to be redundant in several environmental compartments [74, 75], suggesting that fungi in the marine environment are widespread. Sponge-associated bacterial communities, in contrast, are mostly structured by host species [4, 7, 39, 76], but at the same time, they must cover a set of phenotypic biochemical functions [2, 39, 77, 78]. In other words, metabolic redundancy of vastly diversified microbial communities ensures homeostasis and fitness in different sponge holobionts [79, 80]. Sponge mycobiomes in this sense are much less understood, and so are their functional symbiotic capabilities within the host [15, 16].

Information about the symbiotic nature of fungal taxa occurring within sponge hosts is often vague or contradictory [81]. Frequently, microbes transmitted vertically from mother to offspring evoke a sense of beneficial interactions that are guaranteed across generations [82]. While prokaryotic symbionts have been demonstrated to be largely horizontally acquired in some sponges (e.g., *P. ficiformis*) or largely transmitted vertically in others (e.g., *C. crambe* and *C. nucula*)—despite both strategies likely occurring in diverse proportions in all species [39, 83–85]—transmission modes of fungal strains still represent an open question for most sponge holobionts. Vertical transmission tends to promote exclusivity of microbiomes within intraspecific populations, as compared to horizontal acquisition from the environment [82]. In this sense, among our species, yeast generic intraspecific exclusivity was only recorded in *C. nucula* harboring *Schizosaccaromyces* and *Wickerhamomyces* and in *C. crambe* reporting *Genolevuria*. Both of these sponge hosts exhibit predominantly symbiont vertical transmission mode [85, 86], and in the case of *C. nucula*, one of the few works reporting transgenerational acquisition of fungi in Porifera was described in this species [44]. Apart from yeasts, sponges of the genus *Chondrilla* were observed to vertically transmit cyanobacteria (*Synechoccoccus* sp.) to their offspring [87]. Actually, high levels of these latter photosymbionts are required in *Chondrilla* spp. to cover energy budgets [39, 40, 44]. On certain lichenized symbiotic systems, cyanobacteria exhibit increase in photosynthetic potential when engaged with fungi, which convert inaccessible inorganic matter into organic products. Cyanobacteria, in lieu, provide fungi with essential photosynthate carbon and nitrogen sources [88–92]. It could be plausible that in *Chondrilla* sponge holobionts, metabolic demands might be accomplished by cooperating fungi-cyanobacterial consortia, complementing photosymbionts’ inputs [18, 19]. *Crambe crambe* also relies predominantly on vertical acquisition of symbiotic prokaryotic strains [86] and reports a moderate presence of cyanobacteria [39, 84]. At the moment though, vertical transmission of cyanobacteria or fungi has not been documented in this species. Similarly, a symbiotic or nutritional role of cyanobacteria in *C. crambe* has not been clearly determined [39], but as in *C. nucula* above, it could be plausible that this sponge may harbor multi-microbial associations with yeasts, playing cooperative biochemical networks [18–21]. The other host species of the present study had no exclusive yeast genera, and in the case of *P. ficiformis*, horizontal acquisition is the predominant mode of microbial transmission [84, 93, 94], suggesting incorporation of fungal associates from the environment as well.

A common issue in analyzing fungal communities associated with marine invertebrates or environmental compartments is that, still in most cases, a big fraction of the sequences (often > 50%) receives no taxonomic assignments, due to unpopulated and frequently poorly curated and annotated reference databases [38, 74, 75, the present study]. For sponge-fungi associations, the majority of the information on this field comes from cultivation- and isolation-based research [47, 69, 95]. While these techniques are essential to discover new strains with bioactive capabilities, without the combination of metagenomics approaches, they provide an underestimated and biased picture of the sponge mycobiome ecology, as cultivable strains only represent < 1% of the estimated biodiversity [2, 30, 96]. On this matter, Bovio and coauthors (2020) [45] performed a fungal isolation survey on three Mediterranean sponges, finding species-specific mycobiota. The sponge *C. crambe* was investigated and reported to host few fungal strains compared to the other species, leading to the hypothesis that sponge mycobiomes could be shaped by their metabolomes. Indeed, *C. crambe* is known to produce toxic secondary metabolites [97–99] (guanidine alkaloids; Crambescines, Crambescidines), which could limit microbial associations but may also alter the isolation conditions, hampering the growth of some fungal strains [100, 101]. Our findings obtained through a metabarcoding analysis revealed contrasting outcomes. We found sponge mycobiomes lacking species-specific patterns, highlighting stochasticity in their distributions across sponge holobionts and the surrounding seawater. The most abundant fungal genera though (*Aspergillus*, *Cladosporium*, *Pennicillium*, *Malassezia*) were coincident with those from studies performed with different techniques [80, 96, 102]. These findings support the theory that these fungi are widespread regardless marine or terrestrial environment [81]. Only few examples in the literature provide evidence of sponge associated fungi studied with molecular approaches. A pioneer insight into the unculturable mycobiome associated with the Hawaiian sponges was provided by Gao and co-workers (2008) [35]. Using several sets of primers targeting the internal transcribed spacer (ITS), they successfully identified several fungal strains mostly belonging to *Aspergillus*, *Cladosporium*, *Malassezia*, and *Pennicillium*. These findings are consistent with our results on what concerns predominant fungal genera and emphasize that metabarcoding approaches are valuable for disentangling the composition of sponge mycobiomes, highlighting also a much higher diversity obtained as compared to surveys based on strain isolation [71, 81, 103]. More recent studies using solely metabarcoding or combined with cultivation on Mediterranean and North Sea sponges [104], Australian and Mauritius species [81, 103] and Antarctic sponges [105] suggested that fungal-sponge associations are highly variable and can be considered “accidental.” Indeed, a large proportion of fungal sequences was often shared with the surrounding seawater, leading to the hypothesis that these fungi were likely transient and non-symbiotic. Our results are in agreement with these findings, suggesting a predominant horizontal acquisition and transiency of sponge-associated fungi.

Fungal genera commonly associated with sponges, like *Aspergillus*, *Cladosporium*, *Malassezia*, and *Pennicillium*, were reported to possess polyketide synthase and non-ribosomal peptide synthase genes, thus supporting biosynthetic capabilities for bioactive secondary metabolite production with chemical defense or microbial regulation means [28, 106]. In fact, several of these strains exhibited antimicrobial or antiviral activities, suggesting the involvement of these fungi in chemical defense and/or biostatic control of the microbiota within the sponge holobiont [107–110]. Marine fungi have enormous genomic and biochemical versatility in terms of allelochemicals and exoenzymes that could potentially benefit the host to avoid predation, promote nutrient recycling, and deal with environmental pressures [14, 15, 111]. Nonetheless, evidenced functional roles of sponge-associated fungi still remain undiscovered. Sponge-fungi associations require accurate attention and extensive research, especially in what concerns repopulation and reference datasets, the design of more specific primers, and the possibility to better understand the ecological role of the marine fungi, combining biochemical and microscopy techniques. Moreover, it would be timely to conduct histological and quantitative protocols on fungal cells within the sponge holobionts (e.g., FISH, CARD-FISH), to better understand the entity and the localization of the sponge mycobiota. Marine mycology is a nascent discipline, and most aspects are yet to be documented on what regards symbiotic interactions and fungal implications on marine holobionts’ physiology and acclimatization capabilities [15, 112, 113]. Therefore, increasing research employing combined multi-omics’ approaches (e.g., metabolomics, meta-transcriptomics, metagenomics) and culture-dependent methods is imperative to establish a robust fundamental understanding of this kingdom in the marine realm. Such initiatives will sustain developing databases (e.g., www.marinefungi.org) that supplement taxonomy with metabolic profiles, simplifying the study of host-mycobiome associations in the marine environment.

## Supplementary Information

Below is the link to the electronic supplementary material.

ESM 1 (DOCX 3.13 MB)

ESM 2 (XLSX 78.6 KB)

ESM 3 (XLSX 28.3 KB)

## Data Availability

Raw sequencing reads have been deposited in the NCBI Sequence Read Archive (SRA) under the accession number PRJNA1186657.
