# Supplementary material for: Marine Mycobiomes Colonize Mediterranean Sponge Hosts in a Random Fashion
Source: Microb Ecol. 2025 Apr 10;88:25. doi: 10.1007/s00248-025-02523-2 (PMC11985663; doi:10.1007/s00248-025-02523-2)
Supplement: Supplementary file 1 [file 248_2025_2523_MOESM1_ESM.docx]

**Supplementary Material:**

**Marine mycobiomes colonize Mediterranean sponge hosts in a random fashion**

Valerio Mazzella*, Geoffrey Zahn, Antonio Dell’Anno, Laura Núñez Pons*

*^1^ Department of Integrative Marine Ecology (EMI), Stazione Zoologica Anton Dohrn, Ischia Marine Centre, Ischia, 80077, Naples (Italy)*

*^2^ NBFC, National Biodiversity Future Center, Piazza Marina 61, Palermo, 90133, Italy*

*^3^ Biology Department - Utah Valley University. 800 W University Parkway SB243c*

*Orem, UT 84058, USA*

*^4^ Department of Life and Environmental Sciences, Polytechnic University of Marche, Via Brecce Bianche, 60131 Ancona, Italy*

*^5^ Department of Integrative Marine Ecology (EMI), Stazione Zoologica Anton Dohrn, Villa Comunale, 80121 Naples (Italy)*

* corresponding authors:

[valerio.mazzella@szn.it](mailto:valerio.mazzella@szn.it); [laura.nunezpons@szn.it](mailto:laura.nunezpons@szn.it)

**Figure S1. Microbial composition based on the ITS1 marker gene.** Taxa bar plot by sample of the overall dataset reporting microbial composition at the kingdom level associated with the four study sponges and in the surrounding seawater. Non-assigned (NA) and misidentified ASVs represented more than half of the total diversity in average and are grouped in grey.

**Figure S2. Shared fungal ASVs.** Network showing the distribution of shared fungal ASVs among the four sponge species and the seawater. Each dot represents an ASV. Each sponge species and the seawater are distinguished by different by colors.

**Figure S3. Venn diagram.** Number of exclusive and shared ASVs and genera among the four sponge species and the surrounding seawater.

**Table S1.** Statistical tables for alpha-diversity indexes, according to ANOVA test. Df: degrees of freedom, Sum sq: sum of squares, Mean sq: mean of squares, F value: F-statistic, Pr(>F): p-value.

```
A) Shannon index

            Df Sum Sq Mean Sq F value  Pr(>F)
Sponge_Species  4  4.335  1.0839   2.349  0.066
Residuals      53 24.455  0.4614


B) Observed ASVs

               Df Sum Sq Mean Sq F value Pr(>F)
Sponge_Species  4   2894   723.6    2.33 0.0678
Residuals      53  16457   310.5
```

**Table S2.** Statistical tables for the beta-diversity indexes, according to PERMANOVA. Df: Degrees of freedom, SumOfSqs: Sum of squares.

```
A) Bray-Curtis resemblance
               Df SumOfSqs      R2      F p-value
Sponge_Species  4   1.8874 0.07328 1.0477 0.2774
Residual       53  23.8694 0.92672
Total          57  25.7568 1.00000

B) Jaccard resemblance

               Df SumOfSqs      R2      F p-value
Sponge_Species  4   1.9591 0.07268 1.0385  0.241
Residual       53  24.9967 0.92732
Total          57  26.9558 1.00000
```

**Table S3.** Mycobiome dataset. The table is reported in the excel file Table_S3.xlsx. SampleID refers to the label of each sample. Abundance is the relative abundance of each ASV in each sample. Sponge species refers to the contracted scientific name of each sponge species*: Crambe crambe*, *Petrosia ficiformis*, *Chondrosia reniformis*, *Chondrilla nucula*. The last six columns of the dataset rely to the taxonomic rank of each fungal ASV.

**Table S4.** List of exclusive and shared fungal genera of each sponge species and the seawater. This dataset is available in the excel file Table_S4.xslx.
